# The effects of Qigong exercise on sleep quality in older adults: a systematic review and meta-analysis

**DOI:** 10.3389/fpubh.2025.1664055

**Published:** 2025-12-19

**Authors:** Xingjian Xiong, Lu Zhang, Enming Zhang

**Affiliations:** 1 School of Sports Medicine and Rehabilitation, Beijing Sport University, Beijing, China; 2 The Rehabilitation Center of National Sports Training Center, Beijing, China; 3 Key Laboratory of Exercise Rehabilitation Science of the Ministry of Education, Beijing Sport University, Beijing, China

**Keywords:** Qigong, exercise, sleep quality, older adults, systematic review, meta-analysis

## Abstract

**Background:**

Sleep disturbances are common among older adults. While pharmacological treatments may offer short-term relief, they are often associated with adverse effects. Non-pharmacological interventions are thus urgently needed. Qigong, a traditional Chinese practice known for its safety and adaptability, has gained attention as a potential intervention to improve sleep. This study aims to systematically review and meta-analyze the existing evidence regarding the effects of Qigong on sleep quality in older adults.

**Methods:**

Seven databases (PubMed, Cochrane Library, Embase, Web of Science, CNKI, VIP, and Wanfang) were searched for randomized controlled trials (RCTs) published up to October 8th, 2025. The primary outcome was the total score of the Pittsburgh Sleep Quality Index (PSQI) and its subcomponents. The methodological quality was assessed using the Cochrane Risk of Bias tool. Statistical analyses were conducted using Review Manager 5.4 and R version 4.2.0.

**Results:**

We included 15 RCTs involving 1,074 participants. Low certainty of evidence showed that Qigong significantly improved sleep quality compared to control groups, as measured by PSQI total score (MD = −2.47, 95% CI [−3.09, −1.85], *p* < 0.001). Substantial heterogeneity was observed (*I*^2^ = 82.3%). Subgroup analyses showed that Baduanjin demonstrated significant improvement in sleep quality (MD = −2.89, 95% CI [−3.39, −2.39], *p* < 0.001), while Wuqinxi did not (MD = −0.64, 95% CI [−3.74, 2.46], *p* = 0.68). Positive effects were observed in participants with sleep disturbances (MD = −3.30, 95% CI [−4.62, −1.98], *p* < 0.001), depression (MD = −1.96, 95% CI [−3.01, −0.90], *p* = 0.0003), and hypertension (MD = −2.61, 95% CI [−3.02, −2.20], *p* < 0.001). Sensitivity analyses confirmed the robustness of the results. However, the overall certainty of the evidence was rated as moderate to low due to the high heterogeneity and risk of bias in some studies.

**Conclusion:**

Qigong, particularly Baduanjin, may effectively improve sleep quality in older adults. Nevertheless, given the methodological limitations and heterogeneity of the included studies, further high-quality research is needed to validate these findings and inform clinical practice.

**Systematic review registration:**

https://www.crd.york.ac.uk/PROSPERO/view/CRD42024621360, identifier: CRD42024621360.

## 1 Introduction

In recent years, global population aging has emerged as a major public health challenge. Aging is associated with various physiological and psychological changes, including a decline in sleep quality. Compared to younger individuals, older adults typically experience less restorative sleep, are more likely to wake up early, and suffer from fragmented sleep patterns (1, 2). Neuroimaging studies suggest that the global brain network may be altered in older adults with poor sleep quality (3). Additional risk factors such as female gender, depressed mood, and chronic physical illness further contribute to sleep disturbances in this population (4). Moreover, older adults are more likely to experience chronic diseases and mental health disorders (5, 6), which can lead to significant physical, emotional, and financial burdens.

Although pharmacological treatments for insomnia—such as eszopiclone, zolpidem, and suvorexant—may offer short-term benefits, they are often associated with adverse effects, including cognitive impairment, behavioral changes, and an increased risk of falls (7). Due to these risks, particularly in older populations, there is growing interest in non-pharmacological approaches to improve sleep (8). Exercise-based interventions have demonstrated positive effects on sleep quality in older adults (9), with recent systematic evidence further supporting the efficacy of diverse modalities—including aerobic, resistance, and mind-body exercises such as Tai Chi, Qigong, and Baduanjin—in improving sleep quality among older women (10). One such approach is Qigong, a traditional Chinese mind-body exercise that has received increasing attention. In Traditional Chinese Medicine (TCM), “qi” refers to the vital energy that sustains life, while “gong” refers to the cultivation or regulation of this energy through practice (11). The history of Qigong spans over 4,000 years, and the term was officially recognized in China in the 1950s (12). Qigong includes various styles such as Baduanjin, Wuqinxi, Liuzijue, and Yijinjing. It is simple to learn, does not require special equipment, and is well-suited for older adults. According to recent epidemiological studies, the number of Qigong practitioners in the United States has increased steadily over the past two decades (13). Compared to Cognitive Behavioral Therapy for Insomnia (CBT-I), which requires specialist delivery and neuromodulation techniques, which demand equipment and expertise (14), Qigong offers a low-cost, accessible, and culturally accepted intervention suitable for large-scale application among older populations (15, 16).

Qigong has been shown to enhance both cognitive and physical function in older adults (17), and is also recommended for managing sleep-fatigue symptom clusters in individuals with cancer (18). Meta-analyses have demonstrated its effectiveness in improving physical and psychological health outcomes, quality of life, depressive symptoms, and self-efficacy among older adults (16, 19). While prior meta-analyses found that Baduanjin may be a useful complementary therapy for older adults with insomnia (20), few have systematically evaluated Qigong as a standalone intervention. Specifically, no meta-analysis to date has compared the differential effects of qigong styles (e.g., Baduanjin vs. Wuqinxi) on sleep outcomes in this population. Moreover, previous reviews predominantly included English-language trials. This review incorporates a broader body of Chinese-language evidence, offering a culturally contextualized analysis of Qigong's role in sleep enhancement among older adults. Therefore, this systematic review and meta-analysis of randomized controlled trials (RCTs) aims to evaluate the effects of Qigong on sleep quality in older adults. By addressing this knowledge gap, we aim to provide evidence-based recommendations for clinicians and contribute to the development of safe, accessible, and culturally appropriate strategies to enhance sleep health in aging populations.

## 2 Methods

### 2.1 Study protocol

This systematic review and meta-analysis was conducted using Preferred Reporting Items for Systematic Reviews and Meta-Analysis (PRISMA) guidelines (21) and registered with PROSPERO (Registration ID: CRD42024621360), an international prospective registry for systematic reviews. PRISMA checklist was provided in Supplementary materials 1.2.

### 2.2 Criteria for inclusion

The inclusion criteria were formulated in accordance with the PICOS principle.

#### 2.2.1 Types of studies

Randomized controlled trials (RCT).

#### 2.2.2 Types of participants

Participants must be older adults with a minimum age of 60 years. We excluded stroke, unstable angina, compensatory heart failure, Parkinson's, severe mental illness, inability to cooperate, and other severe acute and chronic diseases.

#### 2.2.3 Types of interventions

The interventions of our systematic review adopted Qigong exercise as the main intervention method (no limitation to the style)

#### 2.2.4 Types of comparators

The control group received usual care, normal life, health education or other low-intensity exercise.

#### 2.2.5 Types of outcome measures

The main outcome measure was total scores of subjective measures of sleep outcomes at the end point of the intervention. Sleep quality, sleep latency, sleep duration, sleep efficiency, sleep disturbances, use of sleep medication and daytime function was also analyzed in this review.

### 2.3 Search strategy

Electronic database searches were conducted using Boolean logic operators (“AND”, “OR”) to combine search terms. A representative example of the search strategy is as follows: (Qigong OR Baduanjin OR Wuqinxi) AND (sleep OR insomnia) AND (aged OR Senior OR Older adult). Seven databases, including PubMed, Cochrane Library, Embase, Web of Science, CNKI, VIP, and Wanfang were selected. Articles should be published until October 8th, 2025. We excluded conference abstracts, conference papers, personal communications, and ongoing trials. A detailed search strategy was shown in Supplementary Table 1.

### 2.4 Study selection

All identified studies were imported into EndNote 20. After excluding duplicate studies, two independent reviewers screened the titles and abstracts to select studies that met the inclusion criteria. The full-text versions of the eligible studies were then assessed by the same two reviewers using predefined exclusion criteria. Disagreements were resolved through discussion or by consulting a third reviewer. Data from all included studies were extracted into a pre-designed Excel sheet.

### 2.5 Data extraction

According to the Cochrane Collaboration Handbook, study details were extracted independently by two reviewers as follows: First Author, Year of publication, Country of origin, Sample size (experimental/control groups), Mean age of participants, Proportion of female participants (%), Participants' health status, Intervention measures (experimental/control groups), Intervention doses (in minutes), Intervention frequency/duration, Primary outcomes, Movement standardization and Adherence monitoring. If a study reported outcomes at several time points, we used the longest follow-up.

### 2.6 Methodological quality assessment

We used the Cochrane revised tool to assess the risk of bias in randomized trials to evaluate the methodological quality of RCTs (22). Two independent reviewers rated the quality of the RCTs in the following domains: the randomization method, deviations from the intended interventions, missing outcome data, measurement of the outcome, and selection of the reported result. Within each domain, one or more signaling questions had one of five response options: yes, possibly yes, possibly no, no, and no information. These answers lead to the judgments of “high risk of bias,” “low risk of bias,” or “some concerns.” Disagreements were resolved by a third reviewer.

### 2.7 Data analysis

We performed statistical analyses using Review Manager software 5.4 (Cochrane Collaboration, Oxford, UK) and R version 4.2.0 (R Core Team, R Foundation for Statistical Computing, Vienna, Austria). Since the included studies used the PSQI as the measurement tool, we used the weighted mean difference (WMD) to assess the effect of the intervention. We applied a random-effects model for the pooled analysis to account for potential confounding factors in the included studies. We reported all effect sizes with a 95% confidence interval (CI). We used forest plots to present the study results and assess heterogeneity. Heterogeneity was assessed using the *I*^2^ statistic. The magnitude of heterogeneity was interpreted based on guidelines from the Cochrane Collaboration (23): 0%−40%: May not be clinically significant; 30%−60%: May indicate moderate heterogeneity; 50%−90%: May represent substantial heterogeneity; 75%−100%: Implies considerable heterogeneity. When substantial or considerable heterogeneity was detected (*I*^2^ > 50%), subgroup analyses were conducted to explore the influence of study characteristics, such as qigong style and participants' health status. Additionally, meta-regression was performed to identify potential sources of heterogeneity, with intervention doses, percentage of female participants, and cumulative practice dose (i.e., practice frequency × duration per session) serving as effect moderators. Subsequently, sensitivity analyses were carried out to evaluate the stability of pooled estimates and determine whether any individual study exerted undue influence on the overall effect size (24). Furthermore, publication bias was assessed via funnel plot visualization and Egger's test.

### 2.8 Certainty of evidence

We categorize the certainty of evidence for all reported outcomes as high, moderate, low, or very low using the Grading of Recommendations, Assessment, Development and Evaluations (GRADE) approach (25). GRADE evaluated the certainty of evidence for each outcome by considering five potential downgrading factors: risk of bias, inconsistency, indirectness, imprecision, and publication bias. The default quality of evidence for Randomized Controlled Trials (RCTs) is high. After applying any necessary downgrades based on these factors, we determined the final quality grade for the evidence of each outcome. We rated down imprecision if trials were <400 patients for continuous outcomes (25).

## 3 Results

### 3.1 Literature search

**Figure 1:**
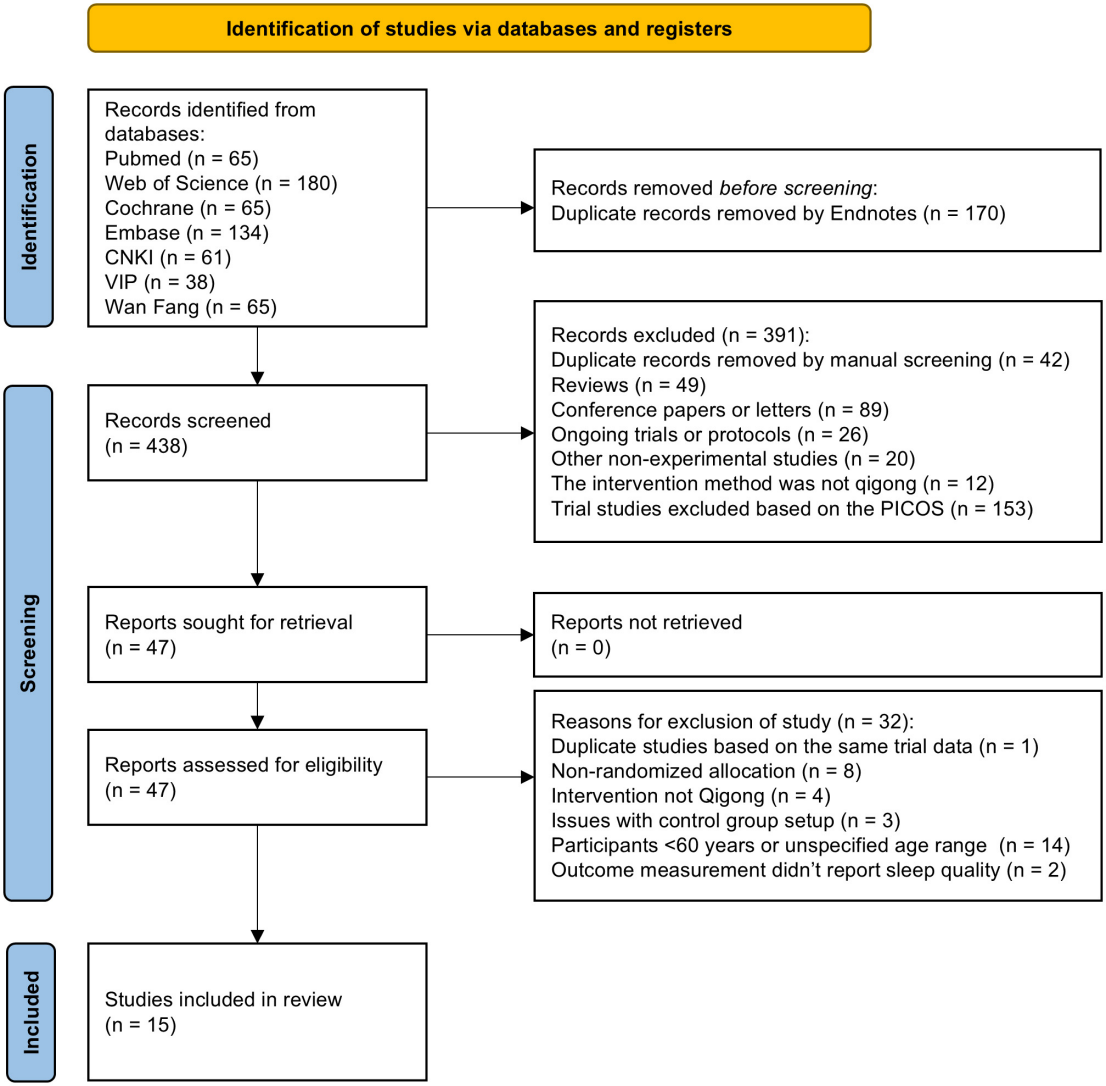
PRISMA flow diagram.

We retrieved a total of 608 records. After removing duplicates and excluding records that did not meet the eligibility criteria, we included 15 studies in the review. The PRISMA flow diagram detailing the study selection process is presented in Figure 1.

### 3.2 Study characteristics

Of the 15 eligible trials (26–40), 14 were conducted in China (26–40), and 1 in Thailand (29). Five studies were published in English (26, 28, 29, 32, 37), while the remaining 10 were in Chinese (27, 30, 31, 33–36, 38–40). Sample sizes ranged from 22 to 72 participants per group, and all studies targeted adults aged 60 years or older. The proportion of female participants varied widely, ranging from 0% to 100%. The studies addressed various health conditions, with three focusing on older adults with sleep disturbances (28, 35, 38), two on depression (29, 40), four on hypertension (30, 31, 33, 36), one on chronic physical illness (32), and one on dementia-related fear (39). Regarding the style of Qigong, most studies used Baduajin (26–28, 30–33, 35, 36, 38, 40), while three studies used Wuqinxi (34, 37, 39). One study did not specify the Qigong style and was therefore not classified (29). Participants in the control group received usual care (26, 28, 30, 34, 36, 37), education (27), medication (31, 33, 40), massage (35, 38), other form of exercise (29) or psychological interventions (32, 39). The frequency and duration of the interventions varied, ranging from 25 to 60 min per day, 2–7 times per week, and lasting from 2 to 26 weeks. Details of the included studies are provided in Supplementary Table 1.

### 3.3 Quality assessment

**Figure 2:**
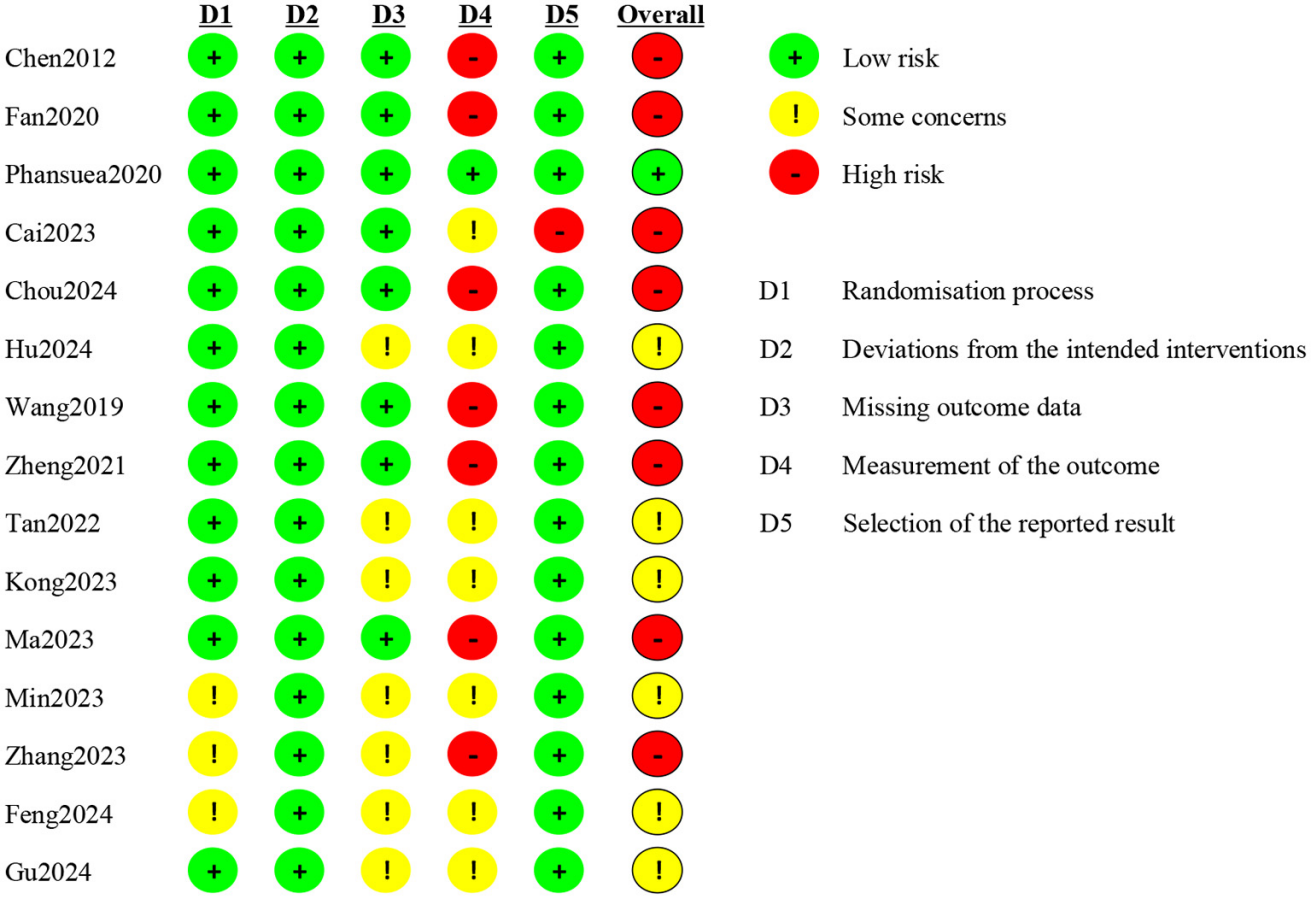
Risk of bias graph: review authors' judgements about each risk of bias item for each included study. (“+”, low risk; “–”, high risk; “!”, some concerns.)

**Figure 3:**
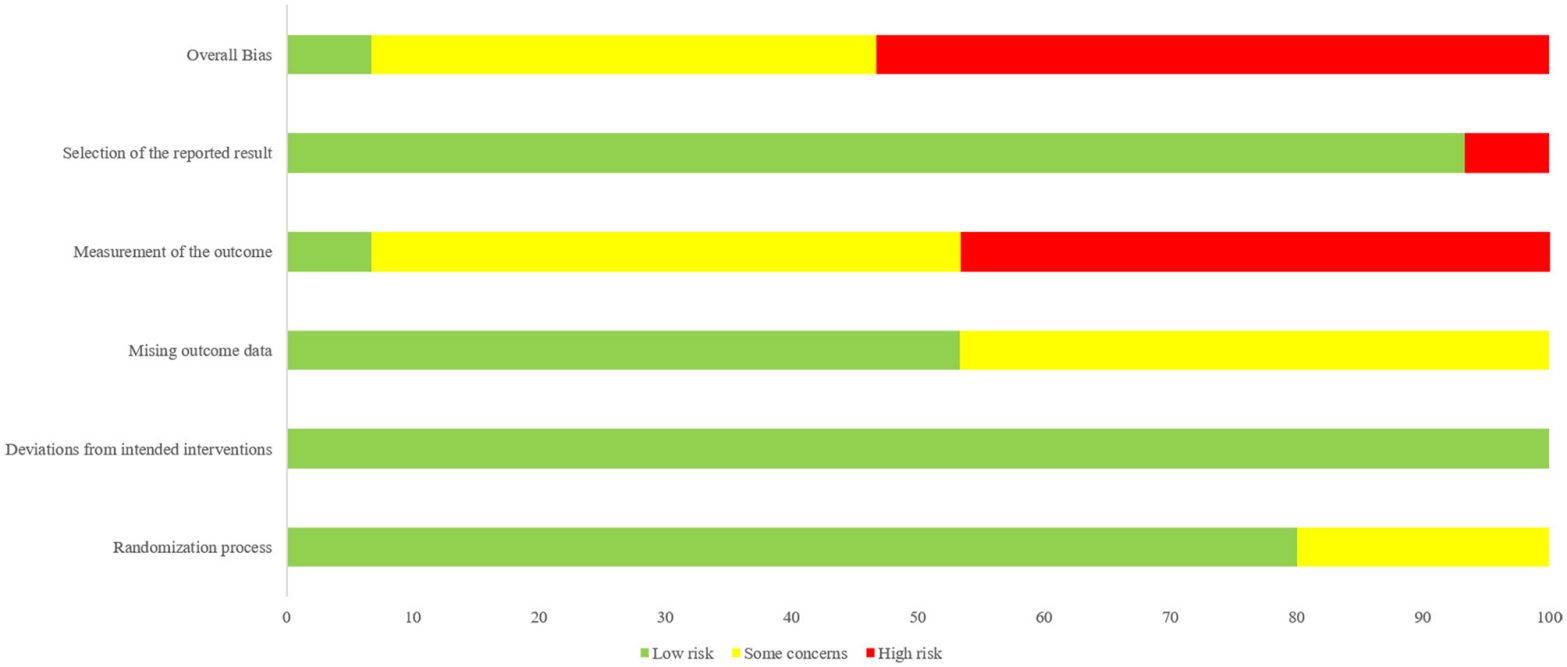
Overall risk of bias: review authors' judgements about each risk of bias item presented as percentages across all included studies.

Of the 15 eligible trials, 14 (93.3%) exhibited a risk of bias in at least one domain (26–28, 30–40). Three studies (20%) did not describe their random sequence generation (35, 36, 38), and none reported deviations from the intended interventions. Seven studies (46.7%) did not mention dropouts (31, 33, 35, 36, 38–40). Only one study included an active control group (29), while seven (46.7%) used passive controls, potentially introducing non-specific factors. One study failed to report the total Pittsburgh Sleep Quality Index (PSQI) score due to an error in the “habitual sleep efficiency” subitem, which may have compromised score reliability (32). Overall, 1 study was rated as having “low risk of bias,” 6 as having “some concerns,” and 8 as having “high risk of bias.” The risk of bias in individual trials was shown in Figure 2. The risk of bias in included studies was demonstrated in Figure 3.

### 3.4 Meta-analysis results

#### 3.4.1 PSQI total scores

**Figure 4:**
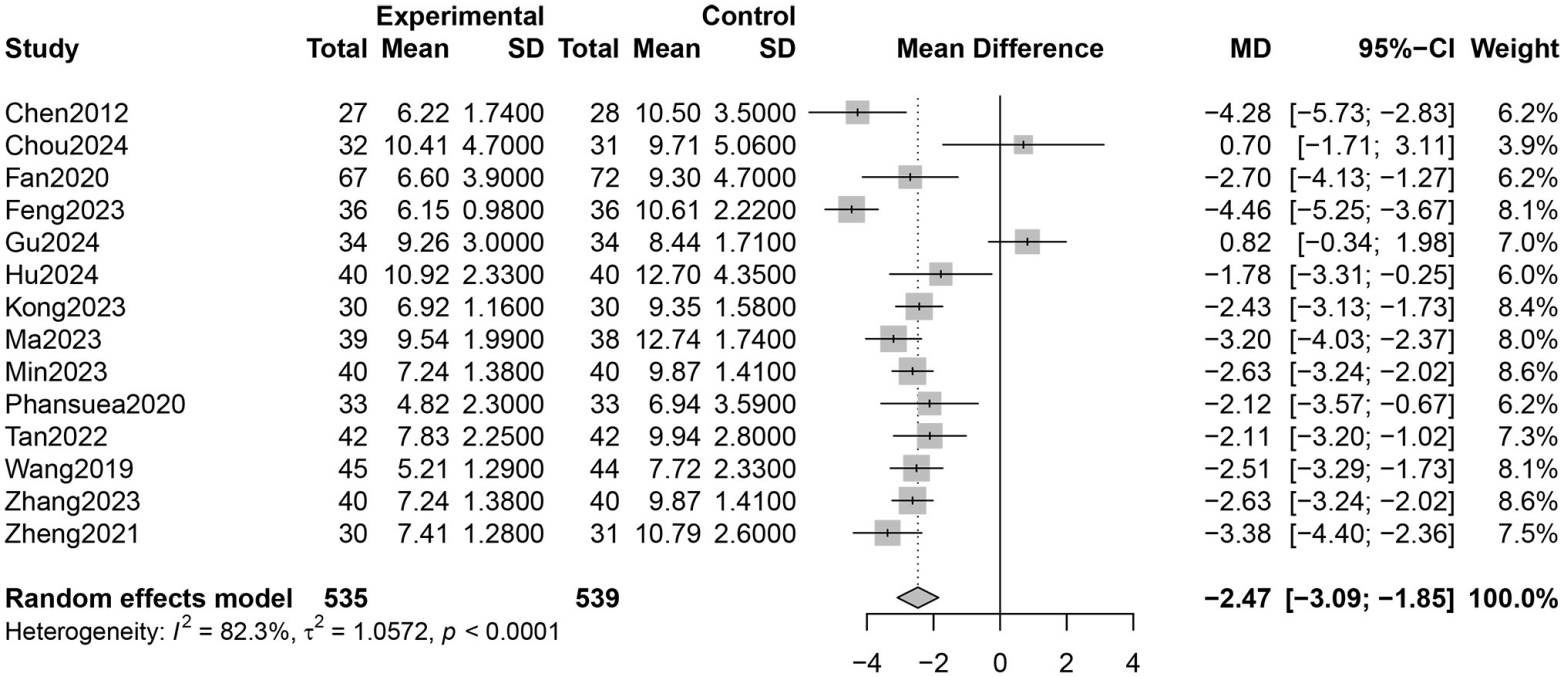
Effect of Qigong on PSQI total scores.

**Figure 5:**
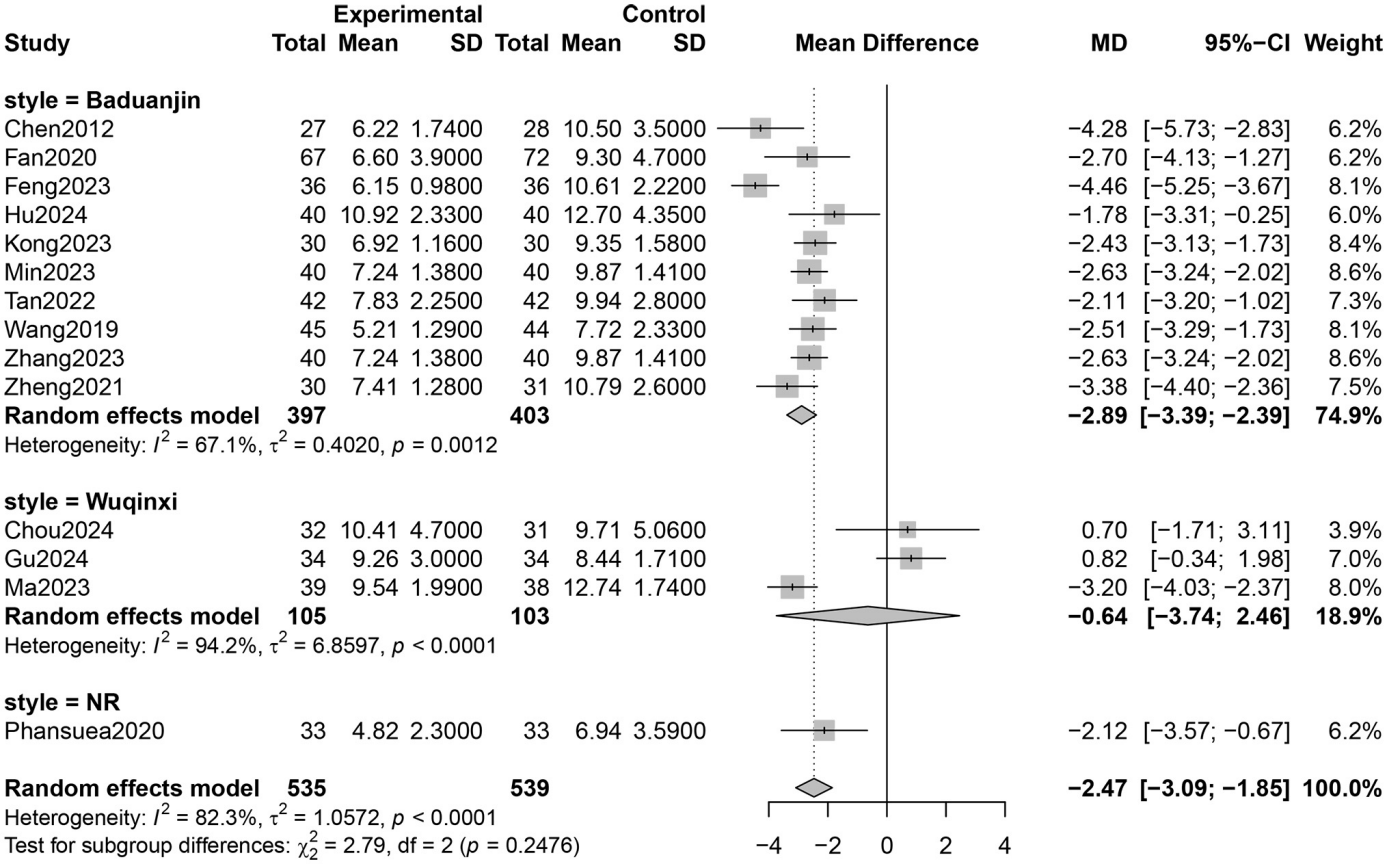
Subgroup analysis according to style of Qigong on sleep quality (NR, not reported).

**Figure 6:**
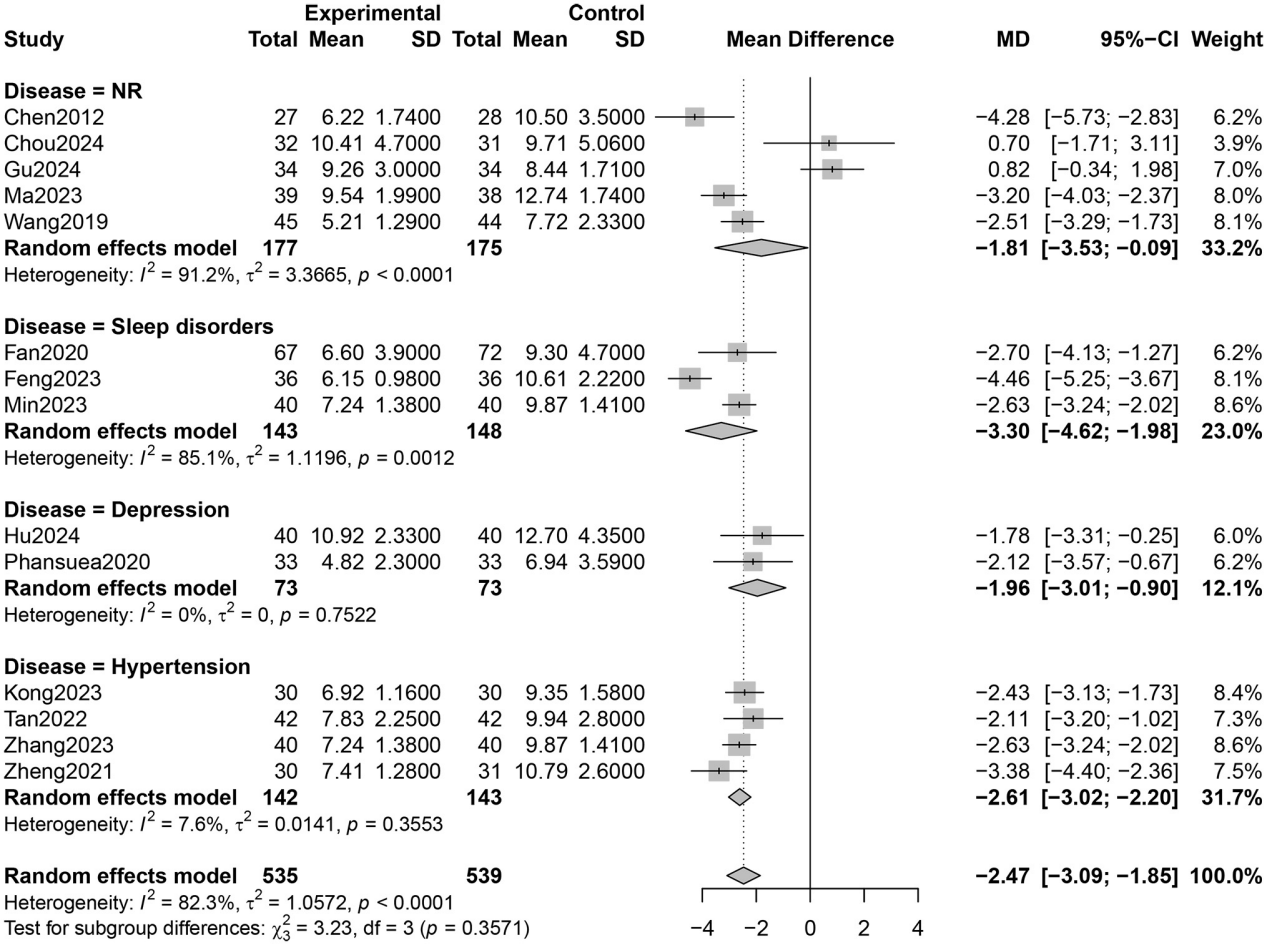
Subgroup analysis according to health status of older adults on sleep quality (NR, not reported).

**Figure 7:**
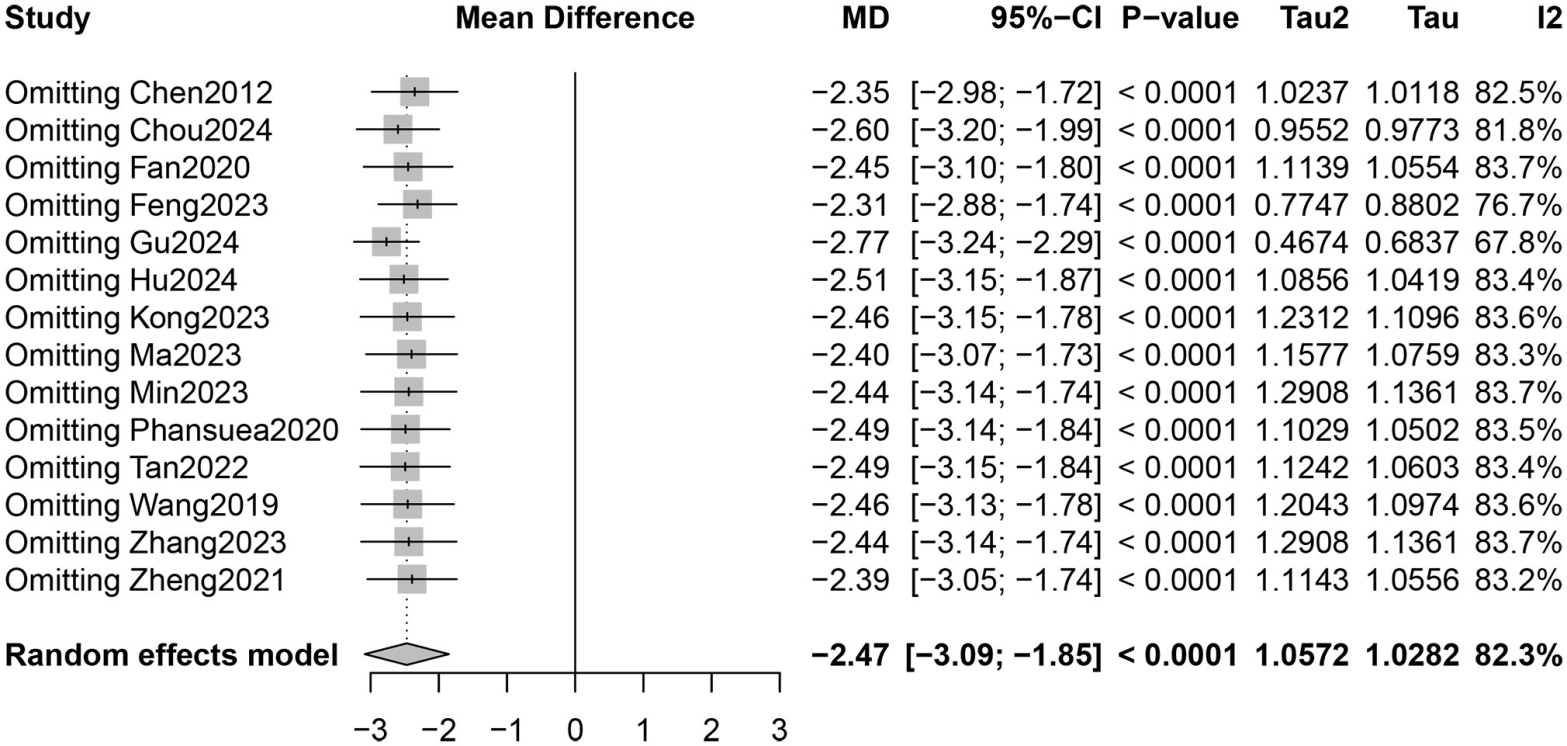
Sensitivity test.

Fourteen RCTs involving 1,027 participants reported PSQI total scores (26–31, 33–40). The certainty of evidence evaluated by GRADE was shown in Supplementary Table 2. Low certainty of evidence showed that Qigong probably improved the sleep quality of older adults compared with control groups (MD = −2.47, 95% CI [−3.09, −1.85], *p* < 0.001; Figure 4). However, high heterogeneity (*I*^2^ = 82.3%, *p* < 0.001) was observed. Subgroup analysis showed that the style of Qigong had different effects on sleep quality. Baduanjin intervention improved sleep quality (MD = −2.89, 95% CI [−3.39, −2.39], *p* < 0.001) and was associated with high heterogeneity (*I*^2^ = 67.1%, *p* = 0.001), while Wuqinxi did not show a significant improvement in sleep quality (MD = −0.64, 95% CI [−3.74, 2.46], *p* = 0.68) and also had high heterogeneity (*I*^2^ = 94.2%, *p* < 0.001) (Figure 5). Additionally, subgroup analysis based on participants' disease conditions showed that Qigong significantly improved sleep quality in older adults with sleep disturbances (MD = −3.30, 95% CI [−4.62, −1.98], *p* < 0.001), depression (MD = −1.96, 95% CI [−3.01, −0.90], *p* = 0.0003), and hypertension (MD = −2.61, 95% CI [−3.02, −2.20], *p* < 0.001). The effect on participants with sleep disturbances was associated with high heterogeneity (*I*^2^ = 85.1%, *p* = 0.001), whereas the depression group (*I*^2^ = 0.0%, *p* = 0.752) and hypertension group (*I*^2^ = 7.6%, *p* = 0.355) showed low heterogeneity (Figure 6). Meta-regression showed that proportion of female, mean ages and total intervention time were not significantly associated with the effect size (Table 1). Sensitivity test demonstrated good robustness (Figure 7). Funnel plot (Supplementary Figure 1) and Egger's test (*t* = 1.09, *p* = 0.295) indicated no potential risk of publication bias.

**Table 1:** Results of meta regression.

**Parameters**	**Coefficient**	***t***	***p***	**95%CI**
Female%	0.05	0.97	0.38	−0.08, 0.17
Age	−0.38	−1	0.36	−1.35, 0.59
Doses	−0.00003	−0.01	0.92	−0.0008, 0.0007

CI, confidence interval.

#### 3.4.2 PSQI sub-item scores

Qigong probably contributed to positive effect on subjective sleep quality (MD = −0.58, 95% CI [−0.76, −0.40], *p* < 0.001, 6 studies, Table 2), sleep latency (MD = −0.53, 95% CI [−0.71, −0.36], *p* < 0.001, 6 studies), sleep duration (MD = −0.44, 95% CI [−0.65, −0.24], *p* < 0.001, 6 studies), sleep efficiency (MD = −0.53, 95% CI [−0.79, −0.27], *p* < 0.001, 5 studies), sleep disturbance (MD = −0.24, 95% CI [−0.32, −0.16], *p* < 0.001, 6 studies), daytime dysfunction (MD = −0.37, 95% CI [−0.46, 0.28], *p* < 0.001, 6 studies), use of hypnotics (MD = −0.12, 95% CI [−0.18, −0.06], *p* = 0.002, 6 studies). All of the outcomes of sub-items were categorized to moderate certainty of evidence except for sleep efficiency (low) (Supplementary Table 2).

**Table 2:** Effect of qigong on sub-items of PSQI.

**Outcomes**	**No of trials**	**MD**	**95%CI**	***I*^2^ (%)**	***Z*-test for overall effect**
Subjective sleep quality	6 (401)	−0.58	−0.76, −0.40	57	*p <* 0.001
Sleep latency	6 (401)	−0.53	−0.71, −0.36	47	*p <* 0.001
Sleep duration	6 (403)	−0.44	−0.65, −0.24	68	*p <* 0.001
Sleep efficiency	5 (356)	−0.53	−0.79, −0.27	80	*p <* 0.001
Sleep disturbance	6 (400)	−0.24	−0.32, −0.16	16	*p <* 0.001
Daytime dysfunction	6 (401)	−0.37	−0.46, −0.28	77	*p <* 0.001
Use of hypnotics	6 (401)	−0.12	−0.18, −0.06	22	*p =* 0.002

MD, mean difference; CI, confidence interval.

## 4 Discussion

This review systematically examined the effectiveness of Qigong in improving sleep quality among older adults. While the overall findings indicate a significant positive effect, the certainty of evidence remains low, primarily due to substantial heterogeneity and the risk of bias in the included studies. Among the Qigong styles, Baduanjin demonstrated the most consistent benefits, whereas Wuqinxi showed limited efficacy, possibly due to the small sample sizes and inconsistent intervention protocols.

Previous reviews have demonstrated the efficacy of Qigong in improving sleep quality across diverse populations. For instance, Qigong has been recommended for breast cancer survivors to enhance sleep quality (41). Additionally, Baduanjin was found to improve physical and mental wellbeing in patients with cardiovascular diseases (42), while traditional Chinese mind-body exercises positively affected sleep quality in individuals with chronic fatigue syndrome (43). Furthermore, our findings are consistent with a recent systematic review, which summarized evidence from randomized controlled trials on the effects of various exercise modalities on sleep quality in older adults, with a focus on older women adults. Their review highlighted that mind-body exercises, including Qigong and Baduanjin, were associated with improvements in sleep latency, efficiency, and overall PSQI scores. While their analysis emphasized older women as a high-risk group, our results extend support for the potential benefits of Qigong in improving sleep quality among the broader older adult population (10). Wu found that both traditional Chinese exercises and general aerobic exercises improved sleep quality in aging people with sleep disturbances [Yang-hao-tian (44)]. Liang found that Baduanjin probably reduced PSQI total and dimension scores, thus improving sleep quality in older adults (20). Furthermore, Ko found that percentage of women and age were significantly associated with the effect size of Qigong on sleep quality (45), while Wu found no relationship, which was in line with current study. Overall, the results of our study were similar to previous meta-analysis, suggesting that qigong may improve sleep quality in the older adults.

Aging is associated with reduced melatonin secretion and disrupted circadian rhythms (46). Insufficient daytime physical activity and a lack of regular exercise were significant contributors to poor sleep quality (47), which may explain why Qigong increased sleep quality in older adults. Moreover, Qigong practice enhances parasympathetic activity and suppresses sympathetic activity through controlled breathing and heart rate regulation (48). This autonomic balance promotes quicker sleep onset and fewer nighttime awakenings, benefiting older adults with hypertension. Older adults are more likely to experience anxiety and depression, which can significantly disrupt sleep (49). Poor sleep, consequently, worsens emotional distress by increasing stress and reducing emotional stability, creating a vicious cycle between sleep problems and mental health issues (50, 51). Qigong helps break this cycle by improving emotional wellbeing and promoting calmness (52), fostering a sense of mindfulness and self-awareness, which enhances resilience to stress and promotes mental wellbeing (53). Furthermore, according to traditional Chinese medicine, Qigong promotes health by regulating the flow of ‘qi' (vital energy) in the body (12). It combines slow movements and controlled breathing to improve circulation, support organ function, and activate the body's natural healing processes. These effects enhance both physical and mental wellbeing (54, 55). When considering Qigong alongside other non-drug sleep treatments, it offers clear practical benefits. Unlike cognitive behavioral therapy for insomnia which requires trained therapists (56), or neuromodulation techniques needing special machines, Qigong doesn't need special equipment or places to practice. Its movements are easy to learn at home or in groups (57). In East Asian communities, people are familiar with this practice (58). Compared to exercises like running or aerobics which might be too hard for frail older adults (59), Baduanjin's gentle movements are safer while still helping relaxation through breathing control (60). This makes it a useful option for older people who need low-risk activities (61).

Despite observing positive effects of Qigong, significant heterogeneity was detected across studies. While subgroup analyses and meta-regression failed to identify significant sources of overall heterogeneity, several unmeasured but methodologically and practically relevant factors may still explain the observed variability. First, methodological flaws across studies introduced inherent variability independent of Qigong itself. As noted in the Methods section, 3 of the 15 included RCTs did not report random sequence generation methods, raising concerns about baseline imbalance between intervention and control groups; 7 studies failed to disclose dropout data. Both issues may overestimate intervention efficacy. More critically, only 1 study used an active control (low-intensity aerobic exercise). This lack of active controls means the observed sleep improvements may partially reflect placebo effects rather than the true therapeutic effect of Qigong, thereby amplifying between-study heterogeneity. Second, inconsistencies in Qigong intervention delivery—particularly in movement standardization and adherence monitoring—further contributed to outcome variability. In terms of movement standardization, some relied on full-time guidance from certificated instructors, others used self-guided learning via videos with no verification of movement accuracy; and a few adopted mixed methods. This variability directly affects intervention fidelity. Adherence monitoring methods also varied widely. Different supervision intensities likely influenced participants' adherence to standardized movements—weaker monitoring (or no monitoring) may lead to inconsistent practice quality, while frequent telephone check-ins could prompt more rigorous adherence—ultimately introducing variability in reported effect sizes. Third, protocol inconsistencies within specific Qigong styles exacerbated subgroup heterogeneity, particularly for Wuqinxi. As documented in previous research (62), variations in core practice parameters—including movement speed (e.g., slow, controlled motions vs. rushed execution), range of motion (e.g., full arm extension vs. partial movement), and animal-mimetic technique details (e.g., the degree of “ape-like” agility in Wuqinxi's ape part)—can significantly alter the intervention's intensity and physiological impact. In our sample, the 3 Wuqinxi studies lacked detailed description in these parameters. Such fundamental protocol differences likely explain the subgroup's extremely high heterogeneity (*I*^2^ = 94.2%) and non-significant sleep benefits, as the intervention's intended therapeutic components were not uniformly delivered.

Our study offers distinct strengths that enhance its contribution to the field. Given Qigong's cultural roots in China, we comprehensively searched three Chinese databases (CNKI, VIP, Wanfang) to capture evidence often overlooked in English-dominant reviews. We further strengthened methodological rigor by exclusively including randomized controlled trials (RCTs) and employing the GRADE framework to evaluate evidence quality. Finally, our analysis of all Pittsburgh Sleep Quality Index (PSQI) subcomponents demonstrates Qigong's broad, positive impact across multiple dimensions of sleep quality in older adults. However, there are limitations to our research. High heterogeneity across studies may be due to differences in Qigong styles, participant health status, and designs of interventions. Most of the included studies were conducted in China, which could introduce cultural or educational biases. Additionally, most of studies fail to set active control. The lack of exercise control groups in many studies makes it difficult to rule out non-specific factors. Many studies did not report long-term follow-up data, which undermines the overall reliability of the findings. Notably, to address these limitations and reduce the variability of results, future research should prioritize conducting high-quality randomized controlled trials with standardized protocols—such as unified specifications for Qigong movements and rigorous adherence monitoring.

## 5 Conclusions

Qigong, particularly Baduanjin, may effectively improve sleep quality in older adults. Nevertheless, given the methodological limitations and heterogeneity of the included studies, further high-quality research is needed to validate these findings and inform clinical practice.

## Data Availability

The original contributions presented in the study are included in the article/Supplementary material, further inquiries can be directed to the corresponding author.
