# Supplementary material for: The effects of Qigong exercise on sleep quality in older adults: a systematic review and meta-analysis
Source: Front Public Health. 2025 Dec 19;13:1664055. doi: 10.3389/fpubh.2025.1664055 (PMC12757222; doi:10.3389/fpubh.2025.1664055)

Supplementary Material

# Supplementary Data

## Search strategies

### Pubmed

("aged"[MeSH Terms] OR "aging"[Title/Abstract] OR "senior*"[Title/Abstract] OR "elder*"[Title/Abstract] OR "geriatr*"[Title/Abstract] OR "old adult*"[Title/Abstract] OR "Old people"[Title/Abstract] OR "Old person"[Title/Abstract] OR "old population*"[Title/Abstract] OR "old citizen*"[Title/Abstract] OR "Old men"[Title/Abstract] OR "Old male"[Title/Abstract] OR "Old women"[Title/Abstract] OR "Old female"[Title/Abstract] OR "older adult*"[Title/Abstract] OR "Older people"[Title/Abstract] OR "Older person"[Title/Abstract] OR "older population*"[Title/Abstract] OR "older citizen*"[Title/Abstract] OR "Older men"[Title/Abstract] OR "Older male"[Title/Abstract] OR "Older women"[Title/Abstract] OR "Older female"[Title/Abstract]) AND ("sleep"[MeSH Terms] OR "sleep*"[Title/Abstract] OR "insomnia"[Title/Abstract]) AND ("qigong"[MeSH Terms] OR "qigong"[Title/Abstract] OR "Qi gong"[Title/Abstract] OR "Daoyin"[Title/Abstract] OR "Dao yin"[Title/Abstract] OR "Chikung"[Title/Abstract] OR "Chi kung"[Title/Abstract] OR "Wuqinxi"[Title/Abstract] OR "Wu qin xi"[Title/Abstract] OR "Liuzijue"[Title/Abstract] OR "Liu zi jue"[Title/Abstract] OR "Baduanjin"[Title/Abstract] OR "Ba duan jin"[Title/Abstract] OR "yijinjing"[Title/Abstract] OR "yi jin jing"[Title/Abstract])

### Web of Science

#1 TS=(aged) OR TS=(aging) OR TS=(Senior*) OR TS=(Elder*) OR TS=(geriatr*) OR TS=("Old adult*") OR TS=("Old people") OR TS=("Old person") OR TS=("Old population*") OR TS=("Old citizen*") OR TS=("Old men") OR TS=("Old male") OR TS=("Old women") OR TS=("Old female") OR TS=("Older adult*") OR TS=("Older people") OR TS=("Older person") OR TS=("Older population*") OR TS=("Older citizen*") OR TS=("Older men") OR TS=("Older male") OR TS=("Older women") OR TS=("Older female")

#2 TS=("qigong") OR TS=("Qi gong") OR TS=("Daoyin") OR TS=("Dao yin") OR TS=("Chikung") OR TS=("Chi kung") OR TS=("Wuqinxi") OR TS=("Wu qin xi") OR TS=("Liuzijue") OR TS=("Liu zi jue") OR TS=("Baduanjin") OR TS=("Ba duan jin") OR TS=("yijinjing") OR TS=("yi jin jing")

#3 TS=(sleep*) OR TS=(insomnia)

#1 AND #2 AND #3

### Embase

#10. #5 AND #6 AND #9 134

#9. #7 OR #8 3,031

#8. 'qigong':ti,ab,kw OR 'qi gong':ti,ab,kw OR 2,630

'daoyin':ti,ab,kw OR 'dao yin':ti,ab,kw OR

'chikung':ti,ab,kw OR 'chi kung':ti,ab,kw OR

'wuqinxi':ti,ab,kw OR 'wu qin xi':ti,ab,kw OR

'liuzijue':ti,ab,kw OR 'liu zi jue':ti,ab,kw OR

'baduanjin':ti,ab,kw OR 'ba duan jin':ti,ab,kw OR

'yijinjing':ti,ab,kw OR 'yi jin jing':ti,ab,kw

#7. 'qigong'/exp 1,724

#6. #3 OR #4 568,956

#5. #1 OR #2 5,329,062

#4. 'sleep*':ti,ab,kw OR 'insomnia':ti,ab,kw 460,672

#3. 'sleep'/exp 354,960

#2. 'aging':ti,ab,kw OR 'senior*':ti,ab,kw OR 1,564,053 8

'elder*':ti,ab,kw OR 'geriatr*':ti,ab,kw OR 'old

adult*':ti,ab,kw OR 'old people':ti,ab,kw OR 'old

person':ti,ab,kw OR 'old population*':ti,ab,kw OR

'old citizen*':ti,ab,kw OR 'old men':ti,ab,kw OR

'old male':ti,ab,kw OR 'old women':ti,ab,kw OR

'old female':ti,ab,kw OR 'older adult*':ti,ab,kw

OR 'older people':ti,ab,kw OR 'older

person':ti,ab,kw OR 'older population*':ti,ab,kw

OR 'older citizen*':ti,ab,kw OR 'older

men':ti,ab,kw OR 'older male':ti,ab,kw OR 'older

women':ti,ab,kw OR 'older female':ti,ab,kw

#1. 'aged'/exp 4,495,170

### Cochrane library

#1 MeSH descriptor: [Aged] explode all trees 285149

#2 (aging):ti,ab,kw OR (Senior*):ti,ab,kw OR (Elder*):ti,ab,kw OR (geriatr*):ti,ab,kw 92827

#3 ("Old adult"):ti,ab,kw OR ("Old adults"):ti,ab,kw OR ("Old people"):ti,ab,kw OR ("Old person"):ti,ab,kw 892

#4 ("Old population"):ti,ab,kw OR ("Old populations"):ti,ab,kw OR ("Old citizen"):ti,ab,kw OR ("Old citizens"):ti,ab,kw 99

#5 ("Old men"):ti,ab,kw OR ("Old male"):ti,ab,kw OR ("Old women"):ti,ab,kw OR ("Old female"):ti,ab,kw 4070

#6 ("Older adult"):ti,ab,kw OR ("Older adults"):ti,ab,kw OR ("Older people"):ti,ab,kw OR ("Older person"):ti,ab,kw 28359

#7 ("Older population"):ti,ab,kw OR ("Older populations"):ti,ab,kw OR ("Older citizen"):ti,ab,kw OR ("Older citizens"):ti,ab,kw 1055

#8 ("Older men"):ti,ab,kw OR ("Older male"):ti,ab,kw OR ("Older women"):ti,ab,kw OR ("Older female"):ti,ab,kw 4849

#9 #1 OR #2 OR #3 OR #4 OR #5 OR #6 OR #7 OR #8 364343

#10 MeSH descriptor: [Sleep] explode all trees 9243

#11 (sleep*):ti,ab,kw OR (insomnia):ti,ab,kw 71678

#12 #10 OR #11 71767

#13 MeSH descriptor: [Qigong] explode all trees 183

#14 (Qigong):ti,ab,kw OR ("Qi gong"):ti,ab,kw OR (Daoyin):ti,ab,kw OR ("Dao yin"):ti,ab,kw 976

#15 (Chikung):ti,ab,kw OR ("Chi kung"):ti,ab,kw OR (Wuqinxi):ti,ab,kw OR ("Wu qin xi"):ti,ab,kw 75

#16 (Liuzijue):ti,ab,kw OR ("Liu zi jue"):ti,ab,kw OR (Baduanjin):ti,ab,kw OR ("Ba duan jin"):ti,ab,kw 637

#17 (yijinjing):ti,ab,kw OR ("yi jin jing"):ti,ab,kw 76

#18 #13 OR #14 OR #15 OR #16 OR #17 1510

#19 #9 AND #12 AND #18 65

### CNKI

(主题：老年人)OR(篇关摘：老年人(精确))OR(篇关摘：老人(精确))OR(篇关摘：老年(精确))AND(主题：睡眠)OR(篇关摘：睡眠(精确))OR(篇关摘：失眠(精确))AND(主题：气功)OR(篇关摘：气功(精确))OR(篇关摘：五禽戏(精确))OR(篇关摘：六字诀(精确))OR(篇关摘：八段锦(精确))OR(篇关摘：易筋经(精确))

### VIP

((((题名或关键词=老年人 OR 题名或关键词=老年) OR 题名或关键词=老人) AND (题名或关键词=睡眠 OR 题名或关键词=失眠)) AND (((((题名或关键词=气功 OR 题名或关键词=导引) OR 题名或关键词=五禽戏) OR 题名或关键词=六字诀) OR 题名或关键词=八段锦) OR 题名或关键词=易筋经))

### Wanfang

(主题:(老年人) or 题名或关键词:(老年人 or 老人 or 老年)) and (主题:(睡眠) or 题名或关键词:(睡眠 or 失眠)) and (主题:(气功) or 题名或关键词:(气功 or 导引 or 五禽戏 or 六字诀 or 八段锦 or 易筋经))

## PRISMA checklist

| **Section and Topic** | **Item #** | **Checklist item** | **Location where item is reported** |
| --- | --- | --- | --- |
| **TITLE** | | |  |
| Title | 1 | Identify the report as a systematic review. | Page1 |
| **ABSTRACT** | | |  |
| Abstract | 2 | See the PRISMA 2020 for Abstracts checklist. | Page 1 |
| **INTRODUCTION** | | |  |
| Rationale | 3 | Describe the rationale for the review in the context of existing knowledge. | Page 2 |
| Objectives | 4 | Provide an explicit statement of the objective(s) or question(s) the review addresses. | Page 2 |
| **METHODS** | | |  |
| Eligibility criteria | 5 | Specify the inclusion and exclusion criteria for the review and how studies were grouped for the syntheses. | Page 3 |
| Information sources | 6 | Specify all databases, registers, websites, organisations, reference lists and other sources searched or consulted to identify studies. Specify the date when each source was last searched or consulted. | Page 3 |
| Search strategy | 7 | Present the full search strategies for all databases, registers and websites, including any filters and limits used. | Page 3, supplementary materials page 1 |
| Selection process | 8 | Specify the methods used to decide whether a study met the inclusion criteria of the review, including how many reviewers screened each record and each report retrieved, whether they worked independently, and if applicable, details of automation tools used in the process. | Page 4 |
| Data collection process | 9 | Specify the methods used to collect data from reports, including how many reviewers collected data from each report, whether they worked independently, any processes for obtaining or confirming data from study investigators, and if applicable, details of automation tools used in the process. | Page 4 |
| Data items | 10a | List and define all outcomes for which data were sought. Specify whether all results that were compatible with each outcome domain in each study were sought (e.g. for all measures, time points, analyses), and if not, the methods used to decide which results to collect. | Page 4 |
|  | 10b | List and define all other variables for which data were sought (e.g. participant and intervention characteristics, funding sources). Describe any assumptions made about any missing or unclear information. | Page 4 |
| Study risk of bias assessment | 11 | Specify the methods used to assess risk of bias in the included studies, including details of the tool(s) used, how many reviewers assessed each study and whether they worked independently, and if applicable, details of automation tools used in the process. | Page 4 |
| Effect measures | 12 | Specify for each outcome the effect measure(s) (e.g. risk ratio, mean difference) used in the synthesis or presentation of results. | Page 4 |
| Synthesis methods | 13a | Describe the processes used to decide which studies were eligible for each synthesis (e.g. tabulating the study intervention characteristics and comparing against the planned groups for each synthesis (item #5)). | Page 4 |
|  | 13b | Describe any methods required to prepare the data for presentation or synthesis, such as handling of missing summary statistics, or data conversions. | Page 4 |
|  | 13c | Describe any methods used to tabulate or visually display results of individual studies and syntheses. | Page 4 |
|  | 13d | Describe any methods used to synthesize results and provide a rationale for the choice(s). If meta-analysis was performed, describe the model(s), method(s) to identify the presence and extent of statistical heterogeneity, and software package(s) used. | Page 4 |
|  | 13e | Describe any methods used to explore possible causes of heterogeneity among study results (e.g. subgroup analysis, meta-regression). | Page 4 |
|  | 13f | Describe any sensitivity analyses conducted to assess robustness of the synthesized results. | Page 4 |
| Reporting bias assessment | 14 | Describe any methods used to assess risk of bias due to missing results in a synthesis (arising from reporting biases). | Page 4 |
| Certainty assessment | 15 | Describe any methods used to assess certainty (or confidence) in the body of evidence for an outcome. | Page 5 |
| **RESULTS** | | |  |
| Study selection | 16a | Describe the results of the search and selection process, from the number of records identified in the search to the number of studies included in the review, ideally using a flow diagram. | Page 5 |
|  | 16b | Cite studies that might appear to meet the inclusion criteria, but which were excluded, and explain why they were excluded. | Page 5 |
| Study characteristics | 17 | Cite each included study and present its characteristics. | Page 5 |
| Risk of bias in studies | 18 | Present assessments of risk of bias for each included study. | Page 6 |
| Results of individual studies | 19 | For all outcomes, present, for each study: (a) summary statistics for each group (where appropriate) and (b) an effect estimate and its precision (e.g. confidence/credible interval), ideally using structured tables or plots. | supplementary materials table 1 |
| Results of syntheses | 20a | For each synthesis, briefly summarise the characteristics and risk of bias among contributing studies. | Page 6 |
|  | 20b | Present results of all statistical syntheses conducted. If meta-analysis was done, present for each the summary estimate and its precision (e.g. confidence/credible interval) and measures of statistical heterogeneity. If comparing groups, describe the direction of the effect. | Page 6 |
|  | 20c | Present results of all investigations of possible causes of heterogeneity among study results. | Page 6 |
|  | 20d | Present results of all sensitivity analyses conducted to assess the robustness of the synthesized results. | Page 6 |
| Reporting biases | 21 | Present assessments of risk of bias due to missing results (arising from reporting biases) for each synthesis assessed. | Page 5 |
| Certainty of evidence | 22 | Present assessments of certainty (or confidence) in the body of evidence for each outcome assessed. | Page 6 |
| **DISCUSSION** | | |  |
| Discussion | 23a | Provide a general interpretation of the results in the context of other evidence. | Page 7 |
|  | 23b | Discuss any limitations of the evidence included in the review. | Page 7,8 |
|  | 23c | Discuss any limitations of the review processes used. | Page 8 |
|  | 23d | Discuss implications of the results for practice, policy, and future research. | Page 8,9 |
| **OTHER INFORMATION** | | |  |
| Registration and protocol | 24a | Provide registration information for the review, including register name and registration number, or state that the review was not registered. | Page 3 |
|  | 24b | Indicate where the review protocol can be accessed, or state that a protocol was not prepared. | Page 3 |
|  | 24c | Describe and explain any amendments to information provided at registration or in the protocol. | Page 3 |
| Support | 25 | Describe sources of financial or non-financial support for the review, and the role of the funders or sponsors in the review. | Page 9 |
| Competing interests | 26 | Declare any competing interests of review authors. | Page 9 |
| Availability of data, code and other materials | 27 | Report which of the following are publicly available and where they can be found: template data collection forms; data extracted from included studies; data used for all analyses; analytic code; any other materials used in the review. | Page 9 |

# Supplementary Figures and Tables

## Supplementary Tables

Table 1. Study characteristics (Exp: experiment group; Con: control group)

| First Author | Year | Country | Sample size (QG/CG) | Mean age (years) | Female (%) | Health status | Intervention(exp/con) | Doses(min) | Frequency/duration | Primary Outcomes | Movement standardization | Adherence monitoring |
| --- | --- | --- | --- | --- | --- | --- | --- | --- | --- | --- | --- | --- |
| Cai | 2023 | China | 25/22 | QG: 68.05 ± 6.59; CG: 67.50 ± 4.32 | 59.6 | chronic physical illness | Baduanjin/cognitive training | 1440 | 60 min/day, 2times/wk, for 12 wk | PSQI, 12,16 wk | Certified instructors | Not mentioned |
| Chen | 2012 | China | 27/28 | QG: 70.48 ± 7.90; CG: 72.96 ± 8.30 | 65.5 | NA | Baduanjin/no intervention | 1080 | 30 min/time, 3 times/wk, for 12 wk | PSQI, 4, 8, 12 wk | Self-guided program | Record form + telephone follow-ups |
| Chou | 2024 | China | 32/31 | QG: 68.81 ± 3.55; CG: 72.39 ± 5.4 | 74.6 | NA | Wuqinxi/no intervention | 1800 | 50 min/time, 3 times/wk, for 12 wk | PSQI, 12 wk | Weekly supervised session plus twice-weekly self-guided practice | Not mentioned |
| Fan | 2020 | China | 67/72 | QG: 70.3 ± 5.7; CG: 71.8 ± 6.7 | 75.5 | sleep disturbances | Baduanjin/no intervention | 5400 | 45 min/day, 5 times/wk, for 24 wk | PSQI, 12, 24 wk | Certified instructors | Not mentioned |
| Feng | 2024 | China | 36/36 | QG: 67.54±4.33; CG: 67.78±4.46 | 44.4 | sleep disturbances | Baduanjin/massage | NA | 45 min/day, for 4 wk | PSQI, 4 wk | Trained by community health workers + instructional videos | Supervised by community medical staff |
| Gu | 2024 | China | 34/34 | NA | NA | dementia fear | Wuqinxi/mindful | 720 | 30 min.day, 3 times/wk, for 8 wk | PSQI, 8 wk | Self-guided program | Not mentioned |
| Hu | 2024 | China | 40/40 | QG: 69.33 ± 5.24; CG: 68.80 ± 5.32 | 100 | depression | Baduanjin/medicine | NA | 10 gradually to 30 min/day, 5times/wk, for 12wk | PSQI, 12 wk | Instructional videos + real-time correction by medical staff | Record form + telephone follow-ups |
| Kong | 2023 | China | 30/30 | QG: 75.53 ± 3.92; CG: 75.47 ± 3.86 | 0 | hypertension | Baduanjin/medicine | NA | 30 min/day, for 2 wk | PSQI, 2 wk | Self-guided program | Not mentioned |
| Ma | 2023 | China | 39/38 | QG: 68.23 ± 5.84; CG: 67.12 ± 5.87 | 62.3 | mild cognitive impairment | Wuqinxi/routine | NA | 2 times/day, 5 times/wk, for 12 wk | PSQI, 4, 12 wk | Certified instructors | Record form + telephone follow-ups |
| Min | 2023 | China | 40/40 | QG: 68.58 ± 3.41; CG: 68.69 ± 3.55 | 48.8 | sleep disturbances | Baduanjin/massage | 1260 | 45 min/day, 7 time/wk, for 4 wk | PSQI, 4 wk | Weekly supervised session + additional self-guided practice | Record form |
| Phansuea | 2020 | Thailand | 33/33 | QG: 69±6.3; CG: 71±6.9 | 72.7 | mild to moderate depression | Qigong/praying and singing | 2160 | 60 min/time, 3 times/wk, for 12 wk | PSQI, 12 wk | Certified instructors | Not mentioned |
| Tan | 2022 | China | 42/42 | QG: 68.93±7.21; CG: 68.95±7.21 | 54.8 | essential hypertension | Baduanjin/medicine | 1800 | 25 min/day, 6 times/wk, for 12 wk | PSQI, 12 wk | Initial instruction by certified instructor + self-guided practice | Telephone follow-ups |
| Wang | 2019 | China | 45/44 | QG: 71.50±8.59, CG: 72.31±8.63 | 55.1 | NA | Baduanjin/education | 2160 | 45 min/day, 4 times/wk, for 12 wks | PSQI. 12 wk | Self-guided program | Telephone follow-ups |
| Zhang | 2023 | China | 40/40 | QG: 67.14±5.01 years; CG: 67.98±4.87 | 41.2 | hypertension | Baduanjin/usual care | 4550 | 35 min/day, 5 times/wk, for 6 months | PSQI, 6 months | Initial instruction by certified instructor + self-guided practice | Record form |
| Zheng | 2021 | China | 34/34 | QG: 70.88 ± 5.56; CG: 70.94 ± 6.34 years | 63.2 | essential hypertension | Baduanjin/usual care | 1560 | 26 min/day, 5 times/wk, for 12 wk | PSQI, 6, 12 wk | Weekly group session + self-guided practice | Record form + telephone follow-ups |

Table 2. GRADE evidence profile of Qigong for older adults

| Outcomes | No of trials | Risk of bias | Inconsistency | Indirectness | Imprecision | Publication bias | Overall quality  of evidence |
| --- | --- | --- | --- | --- | --- | --- | --- |
| PSQI total scores | 14 (1074) | Very serious | No serious inconsistency | No serious indirectness | No serious imprecision | No serious bias | low |
| Subjective sleep quality | 6 (401) | Serious | No serious inconsistency | No serious indirectness | No serious imprecision | No serious bias | moderate |
| Sleep latency | 6 (401) | Serious | No serious inconsistency | No serious indirectness | No serious imprecision | No serious bias | moderate |
| Sleep duration | 6 (403) | Serious | No serious inconsistency | No serious indirectness | No serious imprecision | No serious bias | moderate |
| Sleep efficiency | 5 (356) | Serious | No serious inconsistency | No serious indirectness | Serious | No serious bias | low |
| Sleep disturbance | 6 (400) | Serious | No serious inconsistency | No serious indirectness | No serious imprecision | No serious bias | moderate |
| Daytime dysfunction | 6 (401) | Serious | No serious inconsistency | No serious indirectness | No serious imprecision | No serious bias | moderate |
| Use of hypnotics | 6 (401) | Serious | No serious inconsistency | No serious indirectness | No serious imprecision | No serious bias | moderate |

## Supplementary Figures

Figure 1. Funnel plot


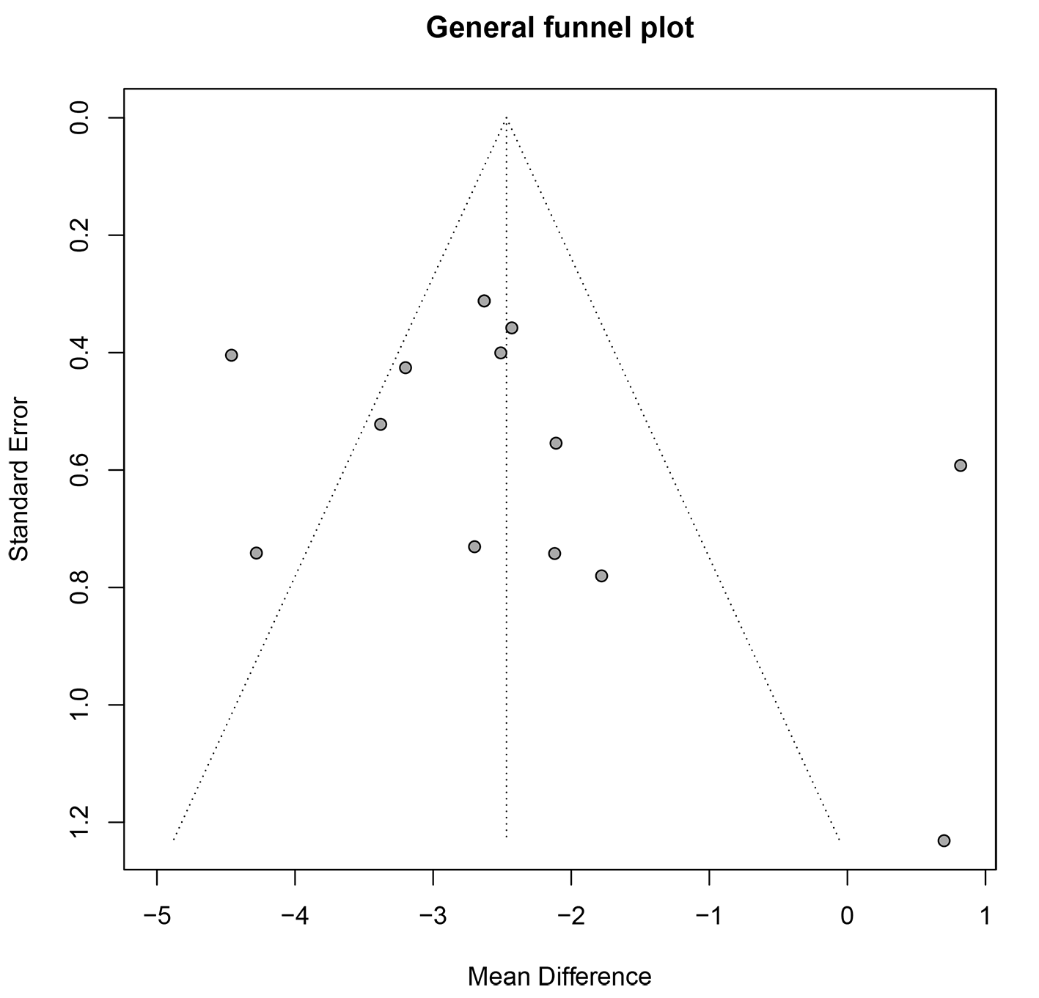

Supplement: Supplementary file 1 [file Table_1.docx]
